# Nurses' Experiences in the ECMO Training Process: A Qualitative Study 

**DOI:** 10.15649/cuidarte.4605

**Published:** 2025-09-01

**Authors:** Marcela Madariaga, Fabian Moreno, John Vergel

**Affiliations:** 1 Nurse, Master’s degree, Universidad del Rosario and Pontificia Universidad Javeriana, Bogotá, Colombia. E-mail: leidy.madariaga@urosario.edu.co Universidad del Rosario and Pontificia Universidad Javeriana Bogotá Colombia leidy.madariaga@urosario.edu.co; 2 Nurse, Master’s degree, Universidad del Rosario and Pontificia Universidad Javeriana, Bogotá, Colombia. E-mail: fabian.morenoc@urosario.edu.co Universidad del Rosario and Pontificia Universidad Javeriana Bogotá Colombia fabian.morenoc@urosario.edu.co; 3 MD, Ph.D. Associate Professor, School of Medicine and Health Sciences, Universidad del Rosario, Bogotá, Colombia. E-mail: john.vergel@urosario.edu.co Universidad del Rosario Bogotá Colombia john.vergel@urosario.edu.co

**Keywords:** Post-Acute COVID-19 Syndrome, Cardiovascular Diseases, Advanced Practice Nursing, Education, Nursing, Graduate, Education, Nursing, Continuing, Extracorporeal Membrane Oxygenation, Síndrome Post Agudo de COVID-19, Enfermedades Cardiovasculares, Enfermería de Práctica Avanzada, Educación de Postgrado en Enfermería, Educación Continua en Enfermería, Oxigenación por Membrana Extracorpórea, Síndrome Pós-Aguda de COVID-19, Doenças Cardiovasculares, Prática Avançada de Enfermagem, Educação de Pós-Graduação em Enfermagem, Educação Continuada em Enfermagem, Oxigenação por Membrana Extracorpórea

## Abstract

**Introduction:**

In 2023, over 20,000 extracorporeal membrane oxygenation (ECMO) therapies were performed worldwide, underscoring the growing need for trained professionals in this intervention. However, standardized ECMO training programs for nurses remain limited and inconsistent.

**Objective:**

To explore professional nurses' learning experiences in ECMO therapy.

**Materials and Methods:**

A qualitative, instrumental case study design was employed with nine ECMO nurses in Colombia, using semi-structured interviews. Thematic analysis was used to identify key themes that emerged from their experiences.

**Results:**

The study revealed considerable variability in ECMO training, which included both formal and informal approaches. Participants reported gaps in theoretical knowledge and practical skills but highly valued the hands-on learning experiences provided by hospitals. Although socio-emotional skills—particularly stress management in critical care settings—were recognized as essential for building confidence, these skills were insufficiently addressed in training programs.

**Discussion:**

While the lack of standardization and the importance of clinical practice and simulation in ECMO training programs are consistent with the existing literature, teaching social-emotional competencies remains an underexplored area.

**Conclusion:**

These findings suggest the need for standardized ECMO educational programs that fully integrate technical, practical, and socio-emotional competencies and address the variability found in both formal and informal educational pathways. Reducing inconsistencies in the educational process could better equip nurses with the confidence to meet the demands of ECMO therapy, ultimately improving patient safety.

## Resumen

**Introducción:**

En 2023 se realizaron más de 20.000 terapias de oxigenación por membrana extracorpórea (ECMO) a nivel mundial, lo que evidencia una creciente demanda de profesionales capacitados para esta intervención. No obstante, estos programas de formación para enfermería siguen siendo limitados y variables.

**Objetivo:**

Explorar las experiencias de formación en la terapia ECMO desde la perspectiva de las enfermeras profesionales.

**Materiales y métodos:**

Se llevó a cabo un estudio de caso instrumental con enfoque cualitativo, utilizando entrevistas semiestructuradas con nueve enfermeros formados en ECMO en Colombia. Se usó análisis temático para identificar los temas clave a partir de sus experiencias.

**Resultados:**

El estudio reveló una gran diversidad en la formación en ECMO, identificando tanto enfoques educativos formales como informales. Las enfermeras reportaron algunas deficiencias en el contenido teórico y en las habilidades prácticas, aunque valoraron el aprendizaje práctico proporcionado por los hospitales. Las competencias socioemocionales, en particular la capacidad de manejar el estrés en situaciones de cuidados críticos, fueron percibidas como cruciales para desarrollar confianza, pero estuvieron poco representadas en los programas de formación.

**Discusión:**

Si bien la falta de estandarización y la importancia de la práctica y la simulación clínica en los programas de formación en ECMO coinciden con la literatura existente, la enseñanza de competencias socioemocionales sigue siendo un área poco explorada.

**Conclusiones:**

Estos hallazgos sugieren la necesidad de programas educativos estandarizados en ECMO que integren las competencias técnicas, prácticas y socioemocionales, y que aborden la variabilidad presente en los programas formales e informales. La reducción de las inconsistencias en el proceso formativo podría preparar mejor a los enfermeros con la confianza necesaria para enfrentar las exigencias de la terapia ECMO, mejorando en última instancia la seguridad del paciente.

## Resumo

**Introdução:**

Em 2023, mais de 20.000 terapias de oxigenação por membrana extracorpórea (ECMO) foram realizadas em todo o mundo, ressaltando a crescente necessidade de profissionais treinados nessa intervenção. No entanto, os programas padronizados de treinamento em ECMO para enfermeiros permanecem limitados e inconsistentes.

**Objetivo:**

Explorar as experiências de aprendizagem de enfermeiros profissionais na terapia com ECMO.

**Materiais e métodos:**

Um estudo de caso qualitativo e instrumental foi conduzido com nove enfermeiros especialistas em ECMO na Colômbia, utilizando entrevistas semiestruturadas. A análise temática foi utilizada para identificar os temas-chave de suas experiências.

**Resultados:**

O estudo revelou considerável variabilidade no treinamento em ECMO, que incluiu abordagens formais e informais. Os participantes relataram lacunas no conhecimento teórico e nas habilidades práticas, mas valorizaram muito as experiências práticas de aprendizagem oferecidas pelos hospitais. Apesar de reconhecer as habilidades socioemocionais — particularmente o gerenciamento do estresse em ambientes de terapia intensiva — como essenciais para a construção da confiança, essas habilidades foram insuficientemente abordadas nos programas de treinamento.

**Discussão:**

Embora a falta de padronização e a importância da prática clínica e da simulação em programas de treinamento em ECMO sejam consistentes com a literatura existente, o ensino de competências socioemocionais permanece uma área pouco explorada.

**Conclusões:**

Esses achados sugerem a necessidade de programas educacionais padronizados em ECMO que integrem plenamente as competências técnicas, práticas e socioemocionais e abordem a variabilidade encontrada nos percursos educacionais formais e informais. A redução de inconsistências no processo educacional poderia dotar os enfermeiros de maior confiança para atender às demandas da terapia com ECMO, melhorando, em última análise, a segurança do paciente.

## Introduction

The COVID-19 pandemic led to the increased use of extracorporeal membrane oxygenation (ECMO) as a life-support intervention for patients with severe respiratory and cardiac failure. As the demand for ECMO therapy rose, the need for qualified professionals to manage this complex therapy became more urgent than ever. This global challenge has stimulated the development and coordination of specialized ECMO centers and educational initiatives around the world.

In 2023, a total of 20,520 ECMO therapies were performed across 593 specialized centers worldwide^1^. The Extracorporeal Life Support Organization (ELSO) plays a key role in organizing and connecting these centers, which are located in countries such as the United States, United Kingdom, Germany, Australia, Canada, France, the Netherlands, Sweden, Spain, Singapore, and several Latin American countries, including Mexico, Brazil, Argentina, Chile, and Colombia. In recent years, several of these countries have begun implementing ECMO education programs. These are offered either through clinical institutions or in partnership with universities. Many of these efforts are supported by international organizations such as ELSO^2^. The goal is to strengthen the preparation of healthcare professionals involved in this therapy.

In Colombia, at least six cardiovascular centers have experience in providing ECMO therapy. Although precise national data on the total number of ECMO procedures performed in 2023 is not available, several institutions have successfully adopted this technology^3^. For example, the Imbanaco Medical Center in Cali performed the country’s first neonatal respiratory ECMO therapy on a patient with a congenital diaphragmatic hernia. Additionally, the Centro de Referencia en Cardiología reported 44 ECMO cases between 2003 and 2011, with an overall survival rate of 64%^4^,^5^. These experiences show the gradual adoption of ECMO therapy in Colombia and highlight the involvement of institutions in developing training initiatives and clinical protocols, often in alignment with ELSO guidelines^3^. 

Before the pandemic, ECMO therapy in Colombia was primarily performed by specialists in perfusion and extracorporeal circulation^6^. However, as the pandemic escalated, professional nurses—often with experience in Intensive Care Units (ICUs)—were rapidly integrated into ECMO care teams to meet the overwhelming demand^3^,^7^. 

While ECMO nurse training existed before the pandemic, the sudden need for more ECMO specialists brought about major changes in the teaching and practice of this therapy^8^. Hospitals and clinics responded by offering specialized training programs and continuing education courses to prepare nurses for the challenges of ECMO care^9^,^10^. Yet, the variability in training programs implemented by healthcare institutions has resulted in a fragmented approach to ECMO training, leaving open the question of which educational strategies are most successful in developing the key competencies needed for ECMO therapy. This issue is particularly relevant, as the success of ECMO therapy relies heavily on the expertise of healthcare professionals and any deficiencies in training can lead to patient safety risks^11^.

Despite the pandemic-driven increase in ECMO use, little research has focused on the learning experiences of nurses trained in ECMO therapy in Colombia or how pandemic-era training programs influenced their competencies. This lack of evidence can lead to discrepancies with international standards, increasing the risk of complications and potential errors in clinical practice. Moreover, the lack of clarity about training methodologies limited our understanding of how well-prepared nurses were to manage this high-risk procedure.

Understanding how these nurses have been trained—and where gaps exist in their training—is necessary, given the critical role ECMO plays in patient survival^12^,^13^. This study aimed to explore the experiences of ECMO specialist nurses regarding their training process, focusing on how they perceived the development of their competencies, the challenges they encountered, and opportunities for improving ECMO education. By understanding these experiences, we can inform future educational strategies to prepare nurses better for the challenges of ECMO therapy.

## Materials and Methods

**Research team**

The research team comprised two professional nurses trained in ECMO therapy. Both nurses were specialists in education for health professionals from the Universidad del Rosario and Pontificia Universidad Javeriana and had one year of experience in qualitative research. The team also included a Ph.D. in education, who provided extensive expertise in research methodology and academic oversight.

**Study design**

Using an instrumental case study design proposed by Stake^14^, this study was conducted within the constructivist epistemological paradigm^15^. A purposive snowball sampling method was used to select participants^16^. First, ECMO nurses known to the researchers were contacted and voluntarily expressed interest in participating; these participants then recommended additional ECMO nurses for the study. In addition, an open call was posted on LinkedIn®, where interested individuals submitted their contact information through a Microsoft Forms® survey. They were then contacted by phone or email and invited to participate in the study. Those who expressed interest received detailed information about the study's aims and procedures and were assured of their right to withdraw from the study at any time. The inclusion criteria required participants to be professional nurses trained in ECMO therapy in Colombia, with a minimum of 2 years' experience in ECMO-related care. Most participants had received their training during the COVID-19 pandemic through either formal academic programs or non-formal institutional courses. At the time of the interview, all participants were actively involved in ECMO care. Efforts were made to ensure a diverse range of professional backgrounds and institutional contexts, including participants from both private and public healthcare institutions. Most participants were based in Bogota and Cundinamarca, with additional representation from other regions of the country. This strategy aimed to capture diverse perspectives on ECMO training, reflecting the diversity of experiences and contexts across Colombia.

Semi-structured interviews were conducted outside of participants' working hours, using the Zoom® platform. The interviews, which averaged 20 minutes in length, were audio-recorded and then transcribed using Microsoft Word 365®'s transcription tool. Researchers then reviewed and edited the transcripts to ensure clarity and organization of the text. Both the audio recordings and transcripts were securely stored in a password-protected location under the custody of the researchers.

Data analysis began with two researchers independently coding key and recurring ideas that emerged from each interview. Thematic analysis^17^,^18^ was conducted, supported by Quirkos® software for data coding and categorization. Preliminary interpretations of codes and categories were then shared with the entire research team. In cases of disagreement about the interpretation of a code or category, the team attempted to reach a consensus. If a consensus was not possible, a third researcher made the final decision. After analysis of the last two interviews yielded no new ideas, the researchers concluded that theoretical saturation had been reached by the ninth interview^19^. The data collected in the interviews is made available for free access and consultation in the research data repository of the Universidad del Rosario^20^.

**Ethical considerations**

This study adhered to ethical standards for research involving human participants in accordance with national and international guidelines. It was classified as low-risk research, and appropriate strategies were implemented to mitigate potential harm. Throughout the research process, the ethical principles outlined in the Belmont Report (respect for persons, beneficence, and justice) were upheld. Prior to participating in the interviews, informed consent was obtained from all participants. The study was reviewed and approved by the Scientific Committee and the Research Ethics Committee of Universidad del Rosario (approval code: DVO005 2502-CV1815).

## Results

The final sample comprised nine ECMO nurses (eight women and one man) who had received training through various educational programs. Most participants were employed in Cundinamarca (n=8), and only one was working in Santander. Eight of the nurses held postgraduate degrees, while one held only an undergraduate degree. On average, the participants had 4.5 years of experience working in ECMO therapy. Regarding training modality, seven participants reported receiving non-formal institutional training, while only two had completed formal programs. The majority of these training programs were initiated in response to the increased demand caused by the COVID-19 pandemic. Two participants obtained certifications issued by universities, while six received institutional certifications from healthcare providers. Each participant was interviewed once, and none withdrew from the study during the process. The demographic and training background details for each participant are presented in Table 1. 

**Table 1:** Participant profiles and training background

Participant	Gender	Region of Employment (Colombia)	Level of profesional training	Time as an ECMO nurse (years)	Type of ECMO training	Training Initiated due to pandemic	University certification	Institutional certification (Health institution)
1	Female	Cundinamarca	Postgraduate	4	Non-formal	Yes	No	Yes
2	Female	Cundinamarca	Postgraduate	4	Non-formal	Yes	No	Yes
3	Female	Cundinamarca	Postgraduate	4	Non-formal	Yes	No	Yes
4	Female	Cundinamarca	Undergraduate	2	Non-formal	Yes	No	No
5	Female	Cundinamarca	Postgraduate	2	Non-formal	Yes	No	No
6	Female	Cundinamarca	Postgraduate	4	Non-formal	Yes	No	No
7	Female	Cundinamarca	Postgraduate	8	Formal	No	Yes	Yes
8	Female	Santander	Postgraduate	4	Non-formal	Yes	No	Yes
9	Male	Cundinamarca	Postgraduate	9	Formal	No	Yes	Yes

Based on the thematic analysis, a total of 23 codes were identified and grouped into four core categories. These categories synthesize the key ideas that emerged from the participants' narratives and provide a framework for presenting their experiences in the following sections.

**Educational processes and limitations in ECMO training**

As the demand for ECMO therapy has grown in recent years, there has been a concomitant need for the creation of specialized ECMO training programs for nurses. While the introduction of new programs provided opportunities for professional growth, it also brought certain obstacles. Initially, ECMO training began informally, with limited opportunities for hospital-based education. These training programs were primarily designed to address the specific care needs of ECMO patients and aligned with established institutional care protocols. Nevertheless, curricula for ECMO training exhibited considerable variability in both theoretical and practical components. The depth of content within the programs was contingent upon the resources available at each institution. The participants noted:

*“... a need arose, and the institution quickly put together a project to meet that need”. (Participant 8).*

*“... luckily, one had the support of a former colleague who kind of gave feedback or solved our doubts”. (Participant 1).*

For example, theoretical instruction, delivered either online or in person, typically covered topics such as cardiopulmonary anatomy and physiology. However, participants often reported gaps in their theoretical knowledge. These subjects were not given the emphasis they expected for a specialty such as ECMO. As a result, participants often had to fill these gaps through independent study, which sometimes led to dissatisfaction. While participants tended to use the terms “feelings” and “emotions” interchangeably, we distinguished between the two for analytical clarity. Emotions were understood as short-term, physiological responses to stressful events, such as fear or anxiety, whereas feelings were considered as longer-lasting, reflective states, such as frustration, satisfaction, or confidence. In addition, clinical practice was considered a highly valuable component of some programs, as participants often engaged in supervised sessions led by an ECMO expert or perfusionist. As one participant noted:

*"...it's not as easy as it seems, but yes, yes, I mean, this hands-on part helped us a lot to make sense of the theory" (Participant 6). *

Support from expert staff was crucial across all training programs, fostering a sense of security and confidence. Participants found the academic workload heavy, particularly given the limited time and pedagogical resources available.

*“…For ECMO therapy, we were referred to the Red Book, which is entirely in English, and not everyone is trained to understand or interpret English properly.” (Participant 9).*

Access to clinical simulation was found to vary according to the resources available at each institution. Those who trained in simulation centers found the experience to be both enriching and a safe introduction to ECMO therapy practice. Other participants reported deficiencies in guided practice prior to working with ECMO patients, frequently due to insufficient feedback or the limited availability of such patients during the training period. The participants shared:

*“... in the practical part, we did it with a simulator, and the simulations were excellent.” (Participant 9).*

*“... it wasn't so easy because I never had the chance to share with an ECMO patient while we were in practice, in theory. Why? Because during that period, those two months, unfortunately, there just weren't any ECMO patient. So, when I got the opportunity, it was much later, and I had to face it on my own.” (Participant 6).*

What began as an informal, hospital-based training program gradually evolved into a more formal educational pathway, which was necessary to certify participants' competencies and clearly define their roles in ECMO therapy. Despite this progress, a lack of access to formal education at universities, along with the high cost and geographic inaccessibility of the few available programs, prevented many nurses from pursuing advanced education in the field.

**Affective dimensions of ECMO therapy training**

The training program evoked a spectrum of emotions and feelings among the participants, particularly regarding the learning process and the educational resources they deemed indispensable for clinical practice. Overall, some participants reported feeling a sense of fulfillment and gratitude for the opportunity to receive training in ECMO therapy and to advance their professional development. Emotions were expressed as immediate reactions, such as fear and anxiety, while feelings referred to longer-lasting states, including satisfaction with teaching methods, like theoretical reviews and hands-on hospital experience during the training period. As one participant noted:

*“... So that was cool. It's nice to be given that kind of autonomy as a nurse to know that you can handle this therapy in this way because you already have the foundation.” (Participant 8).*

Despite the favorable sentiments expressed by some participants, others reported challenges, particularly regarding the superficial treatment of certain theoretical content and the limited time allocated for training. The participants observed that these deficiencies became especially evident when facing real patients, particularly in dealing with unanticipated clinical complications, which frequently resulted in discomfort.

*“... for such specific topics, I would've liked the training to be a bit longer and more demanding.” (Participant 3).*

Some participants expressed frustration with the final assessments of the training programs, describing them as inadequate and overly lenient. This resulted in a sense of dissatisfaction, as many felt that hospitals and clinics often perceived the training process more as an administrative requirement than a genuine advancement in nursing specialization.

*“... I feel like they can just take the exam over and over and over again, and it doesn't really matter; they’ll still pass anyway.” (Participant 3).*

The first experience of caring for an ECMO patient evoked emotions of fear and insecurity in some participants, especially those who felt their training lacked sufficient practical exposure. These emotions were further exacerbated when the participants were confronted with new situations for which they lacked adequate preparation.

*“... going into practice was hard for me. Honestly, I managed, but it was hard because I can tell you, I felt kind of irresponsible doing something I hadn’t done before. So, yeah, there was fear.” (Participant 6).*

In spite of these challenges, the participants exhibited an adaptive capacity, enabling them to face difficulties with enhanced confidence. They acknowledged that it was not feasible to learn all the necessary content during the training program. However, their capacity to apply and integrate both theoretical and practical knowledge enabled them to develop new competencies and effectively manage complex situations in ECMO therapy.

*... you tell yourself, 'Okay, sit down, think, put into practice what you’ve learned, and just get through it.' Maybe you think, ‘Well, this won’t overwhelm me because, after all, it’s a patient.’ We’re healthcare professionals, and there are many things about ECMO you don’t necessarily need to know to handle the situation.” (Participant 6).*

**New ECMO therapy training opportunities arising from the pandemic**

The COVID-19 pandemic marked a turning point in ECMO therapy training for all participants, as it led to an expansion of training opportunities. According to their accounts, the demand for treatment of critically ill patients during the pandemic resulted in increased availability and access to formal and informal training programs in this area. Before the pandemic, some participants indicated that opportunities to train in ECMO therapy were limited, and access to specialized training programs, particularly those offered abroad, was restricted. During the pandemic, many participants observed that the need for more trained ECMO nurses to operate this technology emerged as a pressing concern.

*“...well, I got started because the institution where I currently work had a real need due to the pandemic. The flow of patients was very high and nurses trained in therapy were required but there were no trained personnel at the time”. (Participant 8).*

For those who received training in ECMO therapy during the pandemic, the training methodologies were not perceived as a barrier but rather as an opportunity to expand the scope of training programs. The provision of continuing education in ECMO therapy was not only crucial for improving patient outcomes but also for enhancing the healthcare system's capacity to respond to future health crises. Nevertheless, some participants expressed concerns regarding the efficacy of virtual instruction in an area that demands substantial clinical experience. In particular, some ECMO nurses highlighted the lack of exposure to authentic or simulated practical scenarios as a constraint in developing the clinical competencies essential for managing real-world scenarios involving ECMO therapy. One of the participants explained:

* "...I trained during the pandemic, so the training was quick and I didn’t get to work directly with an ECMO patient unlike many other ECMO nurses who had the opportunity to do it before." (Participant 2)*

**Recommendations for ECMO therapy training**

The participants recommend that ECMO therapy training for new colleagues should integrate not only technical knowledge and clinical skills but also socio-emotional competencies. The training should focus on the combination of theory and practice within a critical care context. Some participants also emphasized that prior experience in ICUs should be a prerequisite for admission to ECMO training programs.

Participants also recommended that ECMO nurses should be prepared to respond to situations requiring a holistic approach, addressing not only the physical but also the emotional and social aspects of patient care. The ability to establish empathetic relationships with patients and their families was considered a crucial skill.

*“...I also feel that the personal aspect, like charisma and attitude, is really important... empathy should be an essential part of the training.” (Participant 6)*

It was also recommended that ECMO therapy training programs should cover a broad range of topics, from cardiovascular anatomy to advanced technologies, and be designed to address the needs of both adult and pediatric patients. Some participants noted that the allocated time for theoretical learning activities was insufficient, which impeded the quality of the learning experience. Enhancing these areas and increasing the time devoted to simulations, such as those involving ECMO pump management, would facilitate comprehensive learning and better prepare nurses to face the clinical challenges of ECMO therapy.

Finally, participants highlighted that the key competencies for ECMO nurses go beyond technical skills as well as the ability to make quick decisions under pressure while always ensuring patient safety:

*“…you need to be a strong leader in these processes... have a clear understanding of the patient's health and condition.” (Participant 3)*

## Discussion

The COVID-19 pandemic has changed the landscape of ECMO therapy, leading to a considerable increase in the demand for this procedure within critical care settings. Prior to the pandemic, ECMO therapy was more commonly used for postoperative patients following cardiovascular surgery or heart and lung transplantation, and the number of qualified ECMO specialists remained relatively limited^3^,^21^. This study reveals that the sudden increase in critically ill patients during the pandemic placed an urgent need on healthcare institutions to train nurses in ECMO therapy rapidly. However, as participants pointed out, the training provided varied widely and lacked standardization in both content and hands-on experience. This lack of standardization could have a negative influence on patient care and clinical outcomes, as nurses may not be uniformly prepared to manage ECMO therapy. While both formal and informal training programs were reported, there were some striking differences in the topics covered and the extent of hands-on training provided^22^.

These findings are consistent with existing literature, such as the recommendations from the Extracorporeal Life Support Organization (ELSO)^19^,^23^, which emphasize the importance of standardizing both the timing and content of training to ensure the acquisition of the competencies required for the role of ECMO specialist. Nevertheless, ELSO also acknowledges that the implementation of such programs must be adapted to the specific needs of each healthcare institution. This flexibility may contribute to inconsistencies in the training process of ECMO personnel.

This tension highlights the challenge of balancing the need for standardized ECMO training with the need to address specific institutional contexts^10^,^24^.

This study shows that hands-on experience is essential in ECMO training, given the procedure's complexity and high-risk nature. Participants emphasized that hands-on experience, especially through high-fidelity simulation, is crucial to developing competence and confidence in managing ECMO patients. Nurses who did not have access to simulations felt more insecure during their initial patient interactions, suggesting that theoretical knowledge alone is insufficient to prepare them for these interactions. These findings also highlight the need to prioritize simulation-based education in ECMO training programs to ensure safe and appropriate patient care. Incorporating simulation can help bridge educational gaps, better prepare nurses for the unique challenges of ECMO therapy, and ultimately improve the quality of care and patient outcomes^9^,^24^,^25^.

Another interesting finding is that ECMO therapy training programs should extend beyond technical instruction to include the development of socio-emotional competencies. Participants emphasized that ECMO nurses must be prepared to manage critical events in the ICU, which requires not only technical expertise but also the ability to manage stress and empathize with patients and their families. Integrating socio-emotional skills into training is imperative for nurses to navigate the emotional challenges of ECMO care^26^,^27^. These findings align with those reported by Hong et al.^12^, who also outlined the importance of comprehensive training that includes both technical and socio-emotional competencies. In addition, prior ICU experience emerged as an important prerequisite, likely because it helps develop key socio-emotional skills necessary for caring for critically ill patients. 

One limitation of this study is the overrepresentation of participants who received informal training, which limited our analysis of those who underwent formal training programs. Formal training refers to training provided by authorized institutions that follow formal curricula and lead to recognized degrees or certifications^28^. In contrast, informal training includes learning experiences designed to complement or update knowledge without following a formal curriculum or leading to a formal credential^28^. While we gained deep insight into the experiences of informal training, the small number of participants with formal training experiences limited our comprehensive understanding of ECMO therapy education. Future research should explore formal training experiences to improve the understanding of educational processes and inform the design of high-quality ECMO training programs.

This limitation highlights the need to standardize training through a systematic, evidence-based approach geared toward continuous improvement. Future studies should also explore the effectiveness of different pedagogical approaches, the impact of socio-emotional support, and the best strategies for preparing nurses in high-pressure environments.

## Conclusions

This study, based on nurses’ learning experiences, reveals that the growing demand for ECMO therapy, especially since the COVID-19 pandemic, has highlighted the urgent need for standardized and in-depth training programs for ECMO nurses. As reported by participants, current training approaches remain inconsistent, particularly in the balance between theoretical instruction and practical experience. Opportunities for improvement include the design of formal curricula tailored to the context of critical care, the development of context-sensitive educational resources, the integration of simulation-based training, and the implementation of continuous assessment strategies. The integration of these elements into ECMO training has the potential to enhance nurses' competence, boost their confidence, and improve their readiness to manage the demands of ECMO therapy while promoting patient safety.
